# A Qualitative Study Exploring the Experiences and Perspectives of Australian Aboriginal Women on Oral Health during Pregnancy

**DOI:** 10.3390/ijerph18158061

**Published:** 2021-07-29

**Authors:** Ariana Kong, Michelle Dickson, Lucie Ramjan, Mariana S. Sousa, Joanne Goulding, Jemma Chao, Ajesh George

**Affiliations:** 1 Centre for Oral Health Outcomes and Research Translation (COHORT), School of Nursing and Midwifery, Western Sydney University, Penrith, NSW 2751, Australia; l.ramjan@westernsydney.edu.au (L.R.); a.george@westernsydney.edu.au (A.G.); 2 South Western Sydney Local Health District, Ingham Institute for Applied Medical Research, Liverpool, NSW 2170, Australia; 3 Sydney School of Public Health, Faculty of Medicine and Health, University of Sydney, Camperdown, NSW 2006, Australia; michelle.dickson@sydney.edu.au; 4 Translational Health Research Institute, Campbelltown, NSW 2560, Australia; 5 IMPACCT—Improving Palliative, Aged and Chronic Care through Clinical Research and Translation, Faculty of Health, University of Technology Sydney, Broadway, NSW 2007, Australia; mariana.desouzaesousa@uts.edu.au; 6 Primary and Community Services, South Western Sydney Local Health District, Liverpool, NSW 2170, Australia; joanne.goulding@health.nsw.gov.au; 7 The Poche Centre for Indigenous Health, University of Sydney, Camperdown, NSW 2006, Australia; jemma.chao@sydney.edu.au

**Keywords:** indigenous, aboriginal, pregnancy, oral health, qualitative, interview, culturally safe, Australia

## Abstract

The aim of this study was to explore whether oral health was an important consideration for Aboriginal and Torres Strait Islander women during pregnancy, whether oral health could be promoted by Aboriginal health staff, and strategies that would be appropriate to use in a new model of care. A qualitative descriptive methodology underpinned the study. All participants in this study identified as Aboriginal, with no Torres Strait Islander participants, and were from New South Wales, Australia. The interviews were analysed using inductive thematic analysis. From the data, two themes were constructed. The first theme identified that oral health was not always the first priority for participants as poor accessibility alongside other competing commitments were challenges to accessing oral health services. The second theme highlighted how relationships with personal networks and healthcare providers were essential and could be used to support maternal oral health during pregnancy. Effective strategies to promote oral health during pregnancy for Aboriginal and Torres Strait Islander women should involve key stakeholders and health care providers, like Aboriginal Health Workers, to facilitate culturally safe support and tailored oral health advice.

## 1. Introduction

Globally, Indigenous pregnant women need culturally safe antenatal support to ensure good health and wellbeing [1,2]. In the Australian context, Aboriginal and Torres Strait Islander peoples experience poorer health outcomes at birth and across the lifespan compared to other Australian peoples [3]. In Australia, Aboriginal and Torres Strait Islander peoples are respectfully referred to as the diverse groups of peoples and nations who were the first custodians and owners of the land [4]. Improving maternal health during pregnancy offers an opportunity to contribute to closing the gap in health inequalities between Aboriginal and Torres Strait Islander children and other children. Maternal oral health is an important area that tends to be overlooked among many women during pregnancy [5]. Antenatal oral health can impact both the mother’s health outcomes and that of her child after delivery [6]. Periodontitis (gum disease) is common among pregnant women [7] and is associated with adverse maternal and delivery outcomes including pre-eclampsia, low birth weight and pre-term birth [6]. Poor maternal dental health is also linked to an increased risk of children having early childhood caries (ECC) and poor oral health over the child’s lifespan [8].

ECC is common among young children, and it disproportionately affects Aboriginal and Torres Strait Islander Australian children compared to other Australian children [9,10]. ECC impacts a child’s quality of life and development, and is also expensive to treat [11]. Yet, ECC is preventable through oral health promotion [11]. Promoting oral health messages that encourage oral hygiene practices, such as tooth-brushing and accessing the dentist, benefits the pregnant woman. Mothers who adopt preventive dental behaviours are likely to teach these practices to their children, which can reduce the risk of ECC [12]. Despite the opportunity to improve this area of inequality for Aboriginal and Torres Strait Islander mothers and children, a recent study found that among Aboriginal and Torres Strait Islander women with young children in one community, none of the women recalled receiving any education or information about oral health during pregnancy [13].

There is the need to promote oral health among Aboriginal and Torres Strait Islander pregnant women, however the legacy of colonisation and intergenerational trauma in Australia continues to be a barrier for many people to engage with government institutions and health services [14]. Aboriginal and Torres Strait Islander women may also experience systemic barriers to accessing dental services [15] including racism [16], and feelings of fear, shame and judgement that result from historical factors [14]. Other barriers to dental care include ineligibility for public dental services, difficulties acquiring a Confirmation of Aboriginality to access Aboriginal community-controlled health services (ACCHSs), high dental costs in a private clinic [14], poor self-efficacy and past dental attendance [17]. In Australia, a Confirmation of Aboriginality refers to a document, typically issued by an Aboriginal community-controlled organisation, certifying that an individual identifies as being of Aboriginal and or Torres Strait Islander descent, and is accepted as being in the community that the person resides or previously resided [18,19]. These documents may be required to access certain Aboriginal community-controlled health services; however, they are also difficult to acquire where family have been removed from their communities because of past assimilation policies [19]. These barriers to dental care highlight that, even if Aboriginal and Torres Strait Islander women receive education about oral health during pregnancy, there remains a need for services to provide continuity and a more coordinated approach to dental care that is underpinned by cultural safety and competence [15].

Currently, many Australian women do not access the dentist during pregnancy [5]. An antenatal oral health program involving trained midwives has been developed and rolled out across Australia in response to the poor awareness and uptake of dental services among pregnant women [20,21]. However, this model of care may not be culturally appropriate nor address the specific needs of Aboriginal and Torres Strait Islander women who may access antenatal care providers other than midwives [22].

Aboriginal and Torres Strait Islander women need to be involved in the development of culturally safe antenatal services that promote oral health during pregnancy if they are to be effective and sustainable [23]. The aim of this study, therefore, was to understand the oral health perceptions and needs for Aboriginal and Torres Strait Islander women during pregnancy. This study specifically explored whether oral health was important for Aboriginal and Torres Strait Islander women during pregnancy, whether oral health could be promoted through Aboriginal health staff inclusive of Aboriginal Health Workers (AHWs) and Family Partnership Workers (FPWs), and strategies that would be appropriate to use in a model of care.

This study is part of a larger program of research, informed by Participatory Action Research (PAR) methodology [24], that aims to pilot test an oral health model of care designed specifically for the community. This larger study was conceptualised with the Aboriginal health staff who support the delivery of antenatal care to Aboriginal and Torres Strait Islander pregnant women in two different Greater Western Sydney (GWS) areas in New South Wales, so that the priorities of Aboriginal and Torres Strait Islander pregnant women would be addressed. The staff identified a need to promote oral health among Aboriginal and Torres Strait Islander women during pregnancy.

There is currently a lack of policy and appropriate interventions to inform a model of care that would address the specific oral health needs for Aboriginal and Torres Strait Islander pregnant women. The Aboriginal health staff specified that to develop a culturally appropriate intervention, focus groups with Aboriginal health staff would need to be conducted followed by yarning with Aboriginal and Torres Strait Islander pregnant women. The focus groups with the Aboriginal health staff, inclusive of AHWs and FPWs, have already been reported elsewhere [14]. Although Aboriginal and Torres Strait Islander is the preferred term to describe the original custodians of Australia, the term Aboriginal will be used to respectfully refer to the women who participated in the study as none of the women identified as Torres Strait Islander.

## 2. Materials and Methods

### 2.1. Qualitative Methodology

A qualitative descriptive methodology underpinned this study to understand the perceptions relating to oral health for Aboriginal and Torres Strait Islander pregnant women. A qualitative descriptive methodology enabled the research team to stay close to the participants’ words and experiences [25]. This was important to ensure an in-depth understanding of socially and culturally constructed phenomena, initially focusing on a literal description which moves beyond into an analysis and interpretation of the meanings participants ascribe and the language they use to shape their realities. This exploratory study aimed to generate new knowledge to inform an oral health model of care, using semi-structured interviews to provide richness to the data. It also allowed exploration of other emerging issues while maintaining a focus on oral health, thereby enhancing both depth and rigor.

Qualitative research approaches have informed interdisciplinary oral health models of care involving the broader health workforce in other populations [26,27,28]. In general, these qualitative studies commenced by identifying existing needs and perspectives of key stakeholders and then explored components of an acceptable, feasible and practical oral health model of care. The current study was conducted using a similar approach which then informed the larger model of care.

### 2.2. Maintaining a Cultural Lens

The lead author (AK) identifies as a non-Indigenous woman with experience in qualitative research. As part of AK’s work as a researcher on the larger study, she has engaged with two local Aboriginal communities in Sydney (NSW) for over two years. AK developed relationships with these communities through yarning with AHWs and FPWs, and by participating in community events.

Ethically, it is essential that Aboriginal and Torres Strait Islander governance guides the research. In this study, Aboriginal researchers with expertise in Aboriginal and Torres Strait Islander research and clinical service delivery were actively engaged in key areas of the research. Aboriginal researchers informed study decision making (JG, MD), informed the data collection process (JG, MD), co-analysed the data and informed the interpretation of the analysis (JC, JG, MD).

### 2.3. Context

This study was based in NSW, where a third (33.3%) of the identified population of Aboriginal and Torres Strait Islander people in Australia reside [4]. Most participants were recruited from a GWS urban area. The GWS area spans a large geographical region across 14 local government areas [29]. Although Sydney is a metropolitan city, some GWS residents live on the rural-urban fringe and have quite different needs to more urban-dwelling residents. Extending recruitment to participants outside the GWS urban areas meant that the perspectives of women in these regions could be included. Compared to people living in GWS urban areas, residents in GWS rural or peri-urban areas generally travel longer distances to access health services that tend to be smaller and have less resources [30,31].

### 2.4. Sampling and Recruitment Strategy

Both purposive and snowball sampling techniques were used to recruit Aboriginal and Torres Strait Islander women who were pregnant or had a child less than a year old. The latter were included as some Aboriginal staff (AHWs and FPWs) were concerned that some women would not have had the opportunity to participate because of other commitments during pregnancy. Women younger than 18 years of age or had high risk pregnancies and were prescribed bed rest by their healthcare provider were excluded.

Participants were recruited for interviews through a flyer posted on the Facebook page of a research centre for Indigenous health (*n* = 2) and by word-of-mouth (*n* = 3). Some participants residing in the GWS area were recruited through antenatal health workers (midwife, AHW, FPW) (*n* = 7). AK provided further information about the study to interested participants and arranged a time and place for the interview. Participants were e-mailed or given a participant information sheet in person, and AK discussed the details of the study with them before obtaining verbal and/or written consent. AK emphasised that all information would be de-identified and confidentiality would be maintained. To build rapport and trust, the interviewer shared about herself and then asked the women about their background, how they identified as an Aboriginal and Torres Strait Islander person (for example, through their mother or father), and the mob or community they identified with.

Participants were recruited until a data saturation was reached [32]; that is, when no new concepts relating to the study aims were identified. Due to the richness and specific focus of the data, the sample size (*n* = 12) was sufficient for this study. A recent systematic review of interview=based qualitative studies found that sample sizes as small as eight or ten are appropriate due to the exploratory nature of the topic area [33].

### 2.5. Demographic Information

The 12 Aboriginal women who participated were between 18 and 36 years of age (median 27.5 years). About half were pregnant (*n =* 7), and in the second trimester (between 14 to 24 weeks’ gestation). All post-partum participants (*n* = 5) had an infant between three and six months of age. Some women (*n* = 8) had up to three other children and almost all women lived with a partner (*n* = 10). Most participants resided in Sydney (*n* = 10). The other two women (Vivian and Belinda) resided in a rural community. Eight participants were employed at the time of the interview. Of the women who were employed, four were on leave. Madison was employed as a case worker, Kelly was a social worker and Ella had a finance and administration role at a not-for-profit organization. Hannah worked part-time at a supermarket whereas Leah had worked in retail casually. Two women, Vivian and Jane, had some experience working as dental assistants whereas Belinda identified as an AHW. Although Audrey was studying for a diploma, she worked previously as an early childhood educator.

### 2.6. Ethical Considerations

Ethical approval for this project was granted by the Aboriginal Health & Medical Research Council (AH&MRC) Human Research Ethics Committee (1438/18) to ensure that the research would be useful, ethical, and valid to Aboriginal communities in NSW. The study was also approved by the South Western Sydney Local Health District (SWSLHD) Ethics Committee (2019/ETH09963), with reciprocal approval granted from Western Sydney University (RH13086). Obtaining approval from both the AH&MRC and SWSLHD human research ethics committees required that the study aligned with the ethical guidelines for research with Aboriginal and Torres Strait Islander Peoples [34]. All participants were given a gift voucher (AUD 50) as reimbursement for their time. Verbally recorded or written consent were obtained from all participants.

### 2.7. Data Collection

All interviews were conducted by AK between August and November 2019. Women were offered the option of either face-to-face or telephone interviews and could bring a support person to the interview. Eight interviews were over the telephone and four were face-to-face. Face-to-face interviews were conducted at a place chosen by each woman, depending on the woman’s needs (at the participant’s home, in a secluded outdoor area and in a private room at a community health centre); light refreshments were also offered. A support person was present in one interview (conducted face-to-face). In another, the interview was held at the participant’s house; a colleague of AK was present as part of the protocol to have two people present for initial home visits. All telephone interviews were conducted in a private room. AK recorded the interviews, and wrote memos during, and immediately after. The semi-structured interviews were between 21 to 77 min. The interview guide can be found in Supplementary Material 1.

### 2.8. Data Analysis

The audio recordings were professionally transcribed, and transcripts were checked by AK. At the conclusion of two interviews, the participants asked AK to censor parts of the interviews. These sections were censored on the transcripts by AK prior to analysis. All participants were de-identified and assigned a pseudonym for confidentiality. The analytical framework described by Braun and Clarke [35] guided the inductive thematic analysis that was used to analyse the data. There are six phases in Braun and Clarke [35]’s framework. As part of the first phase, Familiarising yourself with the data, AK and JC independently read and re-read transcripts to facilitate immersion in the data, each writing memos to capture researchers’ initial thoughts. Memos are a flexible, analytical strategy to assist with interpreting the phenomena shared by the participants [36]. The memos provided AK and JC reflections for further consideration during the thematic analysis process. For the second phase, Generating initial codes, AK created initial codes using NVivo 12 (a qualitative analysis software). AK and JC independently created categories from the initial codes (five and eight, respectively). AK and JC co-constructed five preliminary themes for the third phase, Searching for themes. As part of phase four, Reviewing themes, preliminary themes were discussed with the team. The team then independently reviewed the transcripts and a second meeting was convened to refine the themes until consensus was reached; at that point in the analysis, the five preliminary themes were condensed into two main themes and corresponding sub-themes. This process of refinement and consensus reflected phase five of the framework, Defining and naming themes. For the final phase, Producing the report, the themes and sub-themes were identified, defined and described in this study. The involvement of Aboriginal and Torres Strait Islander researchers was indispensable to ensuring that the Aboriginal subtext (social, cultural and historical context) and assumptions underlying the experiences shared by the participants were meaningfully interpreted through a cultural lens [35].

## 3. Results

From the 12 interviews, the authors constructed two themes, using thematic analysis, relating to oral health during pregnancy (Table 1).

### 3.1. Theme 1: The Priority of Oral Health during Pregnancy

Most women had oral health concerns during pregnancy. Although the participants had different knowledge levels; access and cost of dental services, transport or juggling other responsibilities, meant that the participants prioritised meeting the more basic needs for their family first over oral healthcare during pregnancy.

#### 3.1.1. Oral Health Concerns and Behaviours

Oral health was a concern for most women in this study. Most participants experienced an increase in oral health problems during pregnancy, which either appeared during pregnancy or were exacerbated during pregnancy. Vivian described her experience of this:

*I’ve never had [dental] decay in my life…The only issue I had while pregnant is I got gingivitis, um, and it flared up really bad. And I reckon the minute I gave birth, it all just cleared up.*

These oral health problems included pain, tooth sensitivity, pregnancy gum tumours, bleeding gums and broken teeth. Miranda and Belinda both shared about their oral health problems in pregnancy:

*My oral [health] was really bad. I had a previous drug history, so obviously that rotted a lot of my teeth…The pain was horrible, absolutely horrible.* (Miranda)

*They told me I had pregnancy tumours. My gums were coming away from my teeth. I had good oral hygiene, but I don’t know what happened…They said it stemmed from being pregnant, which made it of course, worse.* (Belinda)

Although some women reported no changes in oral health practices or routines during pregnancy, other participants increased the frequency of regular oral hygiene practices (particularly toothbrushing):

*Same as…pre-pregnancy, which would be brushing my teeth twice a day.* (Kelly)

*Since I’ve fallen pregnant, I don’t like any aftertaste of anything in my mouth, so there’s days where I might even brush my teeth four or five times a day.* (Alice)

The participants also talked about the frequency of dental visits during pregnancy. Although most women accessed a dentist in the past year, some participants reported visiting regularly. Some participants attended the dentist either shortly before, during or after their pregnancy. However, a few women, like Kelly, explained that visits to the dentist were only prioritised if there was an urgent oral health problem:

*I haven’t gone to the doctor—the dentist, in quite a long time and everyone I know don’t go to the dentist—unless it’s to the dental hospital…for emergencies*

#### 3.1.2. Issues with Accessing Dental Services

The participants described various challenges to access dental services to address oral health needs. Some women, like Belinda, described how the waiting list to ACCHS and public dental services were often very long:

*You can’t ring up the hotline and say I’m due for my six-month check and they’ll just basically say all right, you’re on a waiting list, because everyone else that rings up and says I’m in constant pain and I have an abscess, they’ll bump up before ya.*

Vivian, a dental worker, also expressed frustration with the protocols to book a public dental appointment:

*Look, our call centre is no good. It needs to go I personally think. I think we’ve lost a lot of patients because of that. I have contacted that same call centre to make an appointment for my kids to be seen and I waited 40 min…I think we should make our own appointments. It shouldn’t go to a call centre.*

Of the women who saw the dentist, five accessed private dental services, three to public dental services, and one accessed an ACCHS. A few women, like Jane who used to be a dental assistant at a private clinic, were not aware that dental services provided by public or ACCHS were available:

*I actually didn’t even know that there were Aboriginal health clinics…I didn’t know that that was available to us as well.*

Other participants, however, found that certain policies were a barrier to accessing public and ACCHS dental services. Belinda explained that the income she earned during pregnancy made her ineligible for a Health Care Card, a concession card which would enable access to subsidised dental care from the public dental service [37]:

*I wasn’t eligible to go in the public [dental] system because I was working at the time, so therefore I did not have the Health Care Card… [for the public dental service] you have to [be] on either a Health Care Card or Pension Card. So for everyone else—even if you’re working, like you could be working but still be low income, but still not be able to [be put on] cards, you can’t access the dentist.*

Miranda also described how access to the local Aboriginal community-controlled dental clinic required a Confirmation of Aboriginality, which was difficult to obtain.

*I can’t get my [Confirmation of Aboriginality] papers because they can’t track back far enough...*

Few participants described how accessing ACCHS dental services was not always ideal because of the community conflict that can sometimes influence a decision to engage or disengage with an ACCHS. This barrier to accessing services was a described by Vivian:

*If an Indigenous family doesn’t like that family they won’t go to that clinic [managed by that family]. So, I think that’s another massive… they don’t want to walk into that place because they feel like there’s no confidentiality in that workplace.*

A few women both from rural and urban areas also disclosed feeling insecure with confidentiality when attending a service in a close-knit community. Madison spoke of her experience of this:

*Sometimes it’s not good to see someone who’s Aboriginal because we know each other…Communities are small…so sometimes it’s better not to have someone who’s Aboriginal come in and see your—you know, health business.*

The cost of private dental services was also a barrier. Some women, like Belinda and Ella, who accessed private dental services spoke about saving for appointments or using a payment plan to cover the cost of dental procedures:

*The first pregnancy it was a major issue. Like, um, back in 2010, didn’t have much access or even financial—to go the dentists?... I was able to save and save and save money to go to a private dental clinic… [to save took] Probably two years* (Belinda)

*But my dentist does offer a payment plan which is, well, I’m not sure if all of them do, but if you have a procedure done, you can pay off a little bit at a time, which I’ve done that before.* (Ella)

Participants, like Kelly, also managed finances to ensure that essential needs for the family, like food, were met over personal oral healthcare needs:

*I’m about to have a baby and I don’t have $200 to spend. It’s [dental] not covered by Medicare...I would rather buy groceries for the week, quite frankly.* (Kelly)

#### 3.1.3. Knowledge and Attitudes towards Oral Health Care

The participants had different attitudes and levels of knowledge about oral health. Jane and Vivian both worked in a dental setting whereas Belinda identified as a maternal and infant AHW; all three women shared a wealth of oral health knowledge and its link to pregnancy. Vivian spoke of this:

*So basically if you have bad teeth, if you have decay you can be ill, it can make your baby ill.*

Ella, who grew up learning about oral health from her parents, also spoke about the importance of maternal oral health:

*I think it was something that was embedded in me growing up as a child, and I do remember the dentist used to come to our school and do our teeth, so. My parents were always big on protecting your health and your mouth and all that type of thing.* (Ella)

Some women, like Madison, were unsure about the need for, or safety of, dental treatment during pregnancy:

*Like, am I able to go to the dentist? Are they able to rip out teeth? Like do you know what I mean—anaesthesia like all that kind of stuff. What if I need work? Like I have no idea.* (Madison)

There were also some misconceptions about oral health during pregnancy, including that the baby draws nutrients from the mother’s teeth. Hannah described her belief about this:

*I know obviously when you’re pregnant the baby takes a lot from you, so your teeth get very weak… you’ve got to take extra care because they’ve taken all your nutrients and everything.*

Most women, like Alice, also discussed how dentists, or going to the dentist, invoked feelings of anxiety or fear for themselves or the baby:

*I worry a lot ab out going to a dentist and things like that… you just get a bit nervy I suppose.*

For some women, the anxiety stemmed from negative past experiences with dentists. Miranda remembered a fear of dentists stemming back to her childhood:

*I’ve just always had a fear of dentists. I think a lot of it has to do from when I had to get my fillings when I was younger. You know how the dentists go to the schools…they did a bit of a hack job.*

#### 3.1.4. Juggling Competing Priorities

Various participants described various other priorities that needed to be juggled, and sometimes made it challenging to access the dentist. Participants, like Madison, highlighted the difficulties in accessing the dental service due to other commitments:

*Like where do you find the time to go the dentist amongst everything else that you kind of have got on?*

As eight women also had other children, a few mothers spoke about the difficulties of managing children to attend a dental appointment. The lack of time to prioritise a dental appointment was also inferred, particularly if there was limited personal support to supervise the participant’s other children, or restricted opening hours at the dental clinic. Alice and Jane described how they face those challenges:

*I come from such a small town. There’s a lot of convenience there and that, whereas here, it’s a bit of a challenge to get to the supermarket, get the kids in and out of the car and try and get things done and try to get to my appointments and things like that.* (Alice)

*My mum and that finishes early, work, around three [to mind the children]. So, if I made a late appointment I could just leave—later in the day. I suppose it depends on the hours too. How late they’re open...* (Jane)

The time taken to travel to dental appointment can be a concern for women in rural areas. Transport was not a concern for any of the women residing in urban areas. In rural areas, however, the time taken to travel to dental services was a disincentive to attend dental appointments, even when community transport was available. Vivian described this:

*So [towns] are two and a half hours away too… Some of them do come on community transport. But you know what? A lot of those leave at six, seven o’clock in the morning because they don’t just take that one person… So, therefore, I reckon—out of town communities that come to my work, I reckon ninety percent of them don’t show up…*

### 3.2. Theme 2: Supporting Maternal Oral Health Self-Efficacy through Relationships

The participants described how relationships with both healthcare providers and personal networks were beneficial to supporting oral health. Developing trust and connection with healthcare providers was important to tailor oral health advice and enabled participants to make informed oral health choices.

#### 3.2.1. Connection and Trust with Healthcare Providers

The women spoke about various experiences and perspectives on the importance of developing connection and maintaining trust with healthcare providers. These healthcare providers took the time to know the personal needs of the mother and spoke in ways that did not make the mother feel uncomfortable. Participants, like Audrey, also described instances where healthcare providers (including dentists, midwives, doctors or AHWs) had cultivated trust:

*Um, I was—she [midwife] explained stuff really well and the questions that she asked, she didn’t make me feel like it was uncomfortable to speak to her*

Having an Aboriginal healthcare provider was important for some women, like Ella:

*I think the trust and that level of care you can relate to on a cultural basis that you can’t get with other doctors.*

Hannah explained that there was a sense of being welcomed by Aboriginal services that enabled connections to be made more easily with other Aboriginal people:

*I like to stick with the Aboriginal services… Plus, I’ve had them—since basically my daughter was—I was pregnant with my daughter they’ve always been there and helped me.*

Upbringing as a child appeared to influence the connections built with healthcare providers. A few participants, like Alice and Audrey, had maintained relationships with the same healthcare providers from their childhood.

*I’ve grown up going back and forth to the dentist most of my life… I usually go to [the dentist]—where I come from, one back down home* (Alice)

*I see the same doctor I seen as a child at the medical and dental centre* (Audrey)

For other women, like Ella, being able to develop a healthy, trusting relationship with a healthcare provider (including dentists) as a child influenced healthcare access as an adult:

*I always had a dentist that I went to growing up that I really trusted, and I must say I’m scared of dentists. [laughs] So, when I found one, I could really trust, I just stuck with them. Yeah, and it’s just the security of knowing that, okay, I can trust you…*

#### 3.2.2. Seeking Information to Inform Oral Health Choices

Almost all participants spoke about their experiences or attitudes about seeking information or education so they could make informed oral health choices for themselves and for their baby during pregnancy. Vivian and Miranda reflected on the need for oral health education for women during pregnancy:

*I think education is just the biggest key. For people who have bad teeth they don’t realise that what they’re giving their child is bad.* (Vivian)

*But I don’t like needles and I don’t like dentists, [laughs] so I refused to go [to the dentist]. But I had no choice…Like I have to have them [needles]…For the baby, for myself, I need to be alive for my kids. I can’t afford to get sick.* (Miranda)

The information received from antenatal healthcare providers, however, was not consistent across participants, and the difference in quality of information received resulted in different choices being made. For example, Miranda suggested that if she had more information she would have had an earlier visit to the dentist:

*I didn’t think it would hurt the baby...If I would have known that, I would have gone to the dentist a hell of a lot earlier.*

A few women received oral health advice from midwives, as described by Kelly:

*I didn’t even know it [oral health] was important during pregnancy until the midwife told me...They’re not actually one hundred per cent sure why I need to go the dentist but apparently, I do.*

However, other participants took an active role in recruiting health professionals to provide information to others. For example, Belinda spoke about organising dental therapists to provide advice to a mothers group:

*But I’ve had them [dental therapists] come to… a mother’s group [that I run] and explaining about dental issues...*

Where specific advice was not given by a health professional, search engines were used by a few participants to seek information. Ella saw this as problematic, and emphasised that seeking knowledge through a search engine was not ideal due to the conflicting information found online:

*The worst thing I want to do is Google, because they’re saying don’t take this and don’t do this, and the information was conflicting.*

#### 3.2.3. Tailoring Oral Health Promotional Resources

The participants spoke about the need and types of oral health promotional resources and information that would be appropriate. However, almost all participants also highlighted the need for face-to-face education to accompany the resources. This was because in face-to-face education sessions, questions could be answered immediately by the health professional and certain practices could be shown. Hannah and Jane spoke of this preference:

*I think face-to-face is better and obviously if you have questions to ask you can ask there and then.* (Hannah)

*That [face-to-face] was probably more effective, I suppose, compared to any pamphlet you would get. They’re [midwife] able to be there and show you exactly how you should be doing it and, yeah, give you tips as well.* (Jane)

The preferred delivery for advice may also depend on personality types. Sasha, who mentioned that she can get quite shy with face-to-face interaction, preferred to receive advice through text messages:

*I’m a text message sort of person…I get very shy…*

While group education sessions were acceptable for a few participants, other participants preferred to receive education one-on-one. Audrey commented that in some group education sessions, facilitators may put certain people on the spot:

*I’m okay with one on one, but I don’t like group chat...Sometimes I feel like I’m being—you get put on the spot.*

However, Belinda, who was from a rural community, explained that group education sessions could be helpful for some stay-at-home mothers:

*then they [mothers] can say…“I’ve learned all about this oral health”... they’re stay at home mums, and then they come to mothers’ group, it’s getting them out of the house and they want to learn.*

When asked about appropriate oral health resources that could be used to promote oral health, participants identified a range of resources including pamphlets, cards, fridge magnets, SMS reminders, e-mails, dental hygiene products and water bottles with dental messages. Different resources were preferred among the participants:

*You can have a flip chart with a big picture enlarged...* (Belinda)

*Probably even just like a little pamphlet on dental maybe would have been good* (Jane)

*Pamphlets don’t work, I find, because you get so many of them…I find—something that I would use—like dental oral hygiene, water bottles, fridge magnets, things like that* (Kelly)

Participants suggested that the key messages on the resources should include the link between maternal oral health and the baby, the importance of healthy eating, brushing and dental check-ups, and behaviours that should be followed or avoided. Vivian sums up her thoughts on key messages:

*Well, healthy eating, brushing. They would be my two major ones; keep brushing, eat healthy…Regular check-ups…*

The participants also identified the need for more pictures for effective communication, as described by Leah who said, “… *Pictures on things to do and not to do”.*

Including culturally appropriate illustrations to facilitate the delivery of oral health messages on the resources was also considered to be important. Specifically, Belinda recommended the need to use local content that included people who were recognisable to make the resource more culturally appropriate:

*I think local content…try to go to different communities and have local people in the pamphlet, people they can relate to and like, “oh, I know that person. They’re doing it.”* (Belinda)

Some women, like Alice, highlighted that it was essential to have the contact information to direct contact with a dental clinic, to have “*a number that I could call”*.

When asked about using a screening tool, Vivian discussed that integrating a self-administered oral health screening tool on the resource could help pregnant women decide whether access to dental care was needed.

*Screening, yeah. So…“do you have this?” and then a line would say yes or no and if she does go to this, that type of thing?... Yeah, I think that’s good as long as you keep it simple, not heaps of words… I reckon keep it simple, a simple picture and yes or no’s.*

## 4. Discussion

This is the first study to extensively explore the oral health needs and perceptions of Australian Aboriginal women during pregnancy. This study also filled a gap in knowledge about the potential role of the broader health workforce in promoting antenatal oral health care and identified key supportive strategies to inform a culturally safe model of care. Importantly, this study highlights several barriers many pregnant Aboriginal women face in managing oral health. These include barriers previously identified in other studies with Indigenous pregnant women across the globe, such as the need to prioritise time and money on family needs and other responsibilities over personal oral health needs [37], cost of private dental services [13,15], long waiting lists [15], and a lack of eligibility for subsidised public and Aboriginal community-controlled dental services [14,38]. The findings from this study, however, also report other barriers for Australian Aboriginal women during pregnancy, including a lack of information about subsidised public and Aboriginal community-controlled dental services, protocols around booking an appointment at public dental services, and concerns around confidentiality in close-knit communities.

Numerous strategies were suggested to address these barriers and improve oral health of Aboriginal women during pregnancy. These strategies included developing a range of oral health promotion resources that are culturally appropriate and obtaining timely information about the impact of oral health on pregnancy. The findings found that information needs to be delivered face-to-face from a dental or other antenatal care provider that has an established connection with the Aboriginal woman. These strategies need to be considered when developing an oral health model of care for Aboriginal women.

An established connection and trust were essential in the promotion of oral healthcare by healthcare providers. The findings from this present study further indicate that relationships are important to support oral health and oral health self-efficacy. Sources of self-efficacy, which can impact on health behaviours and outcomes, includes encouragement from other people [39]. Australian research by Jamieson, et al. [40] and Ben, et al. [41] show that self-efficacy and possessing a sense of control are associated with improved oral health practices and self-rated oral health among Aboriginal pregnant women. Some participants drew on trusting relationships with healthcare provider to seek health advice. Thus, to discuss oral health within this context, healthcare providers need to establish rapport with clients. A previous study conducted with Aboriginal health staff identified that oral health can be a sensitive issue to discuss with some women [14]. From the perspectives of the participants, however, trust with a healthcare provider could mitigate some of the discomfort associated with discussing oral health problems.

Some women commented on the advantages of receiving care from ACCHSs and Aboriginal healthcare professionals. These advantages were attributed to feeling welcomed and being able to build relationships more easily with another Aboriginal and Torres Strait Islander person. Thus, Aboriginal health staff have a vital role as they possess a wealth of knowledge about culture and social practices and are likely to have culturally safe, effective communication skills to reach, connect and build trust when discussing oral health [42]. Walker, et al. [43] found that Indigenous Health Workers in remote Australia also recognised their potential role in promoting oral health as part of general health. The findings from this study found that some women were worried about confidentiality when disclosing oral health problems to Aboriginal health staff due to the close-knit nature of some communities. Although Aboriginal health staff endeavour to protect professional and personal relationships [44], some Aboriginal women may prefer to access non-ACCHSs. This implies that non-Indigenous health staff and services need to have the appropriate oral health training and support to ensure that Aboriginal women continue to receive culturally safe care. Non-Indigenous health staff also need to learn about how to best build trust, rapport and communicate in ways that are culturally appropriate.

Another key strategy for a model of care is to ensure that Aboriginal women have reliable and tailored oral health information. A review by McCalman, et al. [45] observed the importance of health promotion tools that are disseminated through facilitated implementation by Aboriginal and Torres Strait Islander people, rather than passive distribution. In an antenatal model of oral healthcare, passive dissemination of oral health resources or information would likely not be adequate. The participants in this study wanted to make choices based on quality advice given by an antenatal care provider. Some women received little or inconsistent information about oral health during pregnancy, and had to turn to online sources of information which are often unreliable. The lack of information given to Aboriginal women about oral health and pregnancy has also been identified in another Australian study [13]. The findings from the present study demonstrate the need for antenatal care providers to discuss oral health, which is recommended in Australian pregnancy care guidelines [46]. Antenatal care providers need to discuss oral health in relation to the client’s personal needs and circumstances and identify strategies that could mitigate any challenges faced. Tailored oral health education and support may include timely oral health advice, advice on experiencing dental anxiety and information on accessing affordable dental services.

Based on the findings from this study, a potential culturally safe model of oral healthcare would involve the delivery of oral health and pregnancy advice by an antenatal care provider that the client trusts, such as an AHW, alongside culturally appropriate oral health promotion resources. Unfortunately, this proposed model of care would be limited by barriers like cost of dental treatment, which has also been found to be a barrier in other studies involving Indigenous pregnant women around the world [13,14,15]. As discussed in an earlier study with AHWs [14] and seen in our findings, the cost of dental care can still be significant for women who are employed but have other financial commitments, even though they may have an awareness about the importance of good oral health care. The findings from our study also identified a concern with the process of booking a public dental appointment, which can be another barrier to accessibility for some women. These factors continue to highlight the need for more pragmatic options for culturally safe dental care among Aboriginal and Torres Strait Islander women during pregnancy, such as changes in policy to provide oral health checks for all women during pregnancy [14] and streamlining appointment bookings as identified in this study. Furthermore, there is a need to capacity build AHWs and other antenatal care providers in promoting oral health among pregnant women, and to develop culturally appropriate oral health promotion resources.

The findings highlight several implications for policy and practice. Firstly, there is a need to focus on developing culturally safe oral health training for a large range of antenatal care providers, including AHWs, who are in contact with Aboriginal and Torres Strait Islander women during pregnancy. The way that dental services are delivered also need to be reconsidered. From booking appointments to post-treatment care, dental services need to be adapted to be more culturally safe. Existing external public dental and ACCHS policies also necessitate change to meet the oral health needs of Aboriginal and Torres Strait Islander women who may not qualify for affordable dental care at a time where more support may be required.

## 5. Limitations

There were some limitations to this study. Most of the participants resided in an urban rather than rural or remote area so the experiences shared from a rural perspective were limited. Most interviews were conducted over the telephone which may have affected openness and the interviewer’s ability to read non-verbal cues. For some people; however, anonymity over the phone can enhance the amount of information shared with an interviewer [47,48]. Interviews that were over the phone were on average longer (45.6 min) compared to interviews face-to-face (29.5 min), and also explored the same topics in detail. Moreover, the participants who resided in an urban area could choose to have a face-to-face or telephone interview, depending on the woman’s personal convenience and preference.

Future research needs to explore the oral health perspectives of Aboriginal and Torres Strait Islander pregnant women living in other settings. As this was an exploratory study, larger cross-sectional surveys may also provide more insight into the factors that impact the oral health of Aboriginal and Torres Strait Islander women during pregnancy.

The interviewer also did not have a relationship with the Aboriginal participants prior to the interviews, which could have affected the participants’ openness in sharing experiences or stories. Many participants were recruited through an antenatal health provider with whom they had established a relationship, which also contributed to the level of trust with the interviewer. Two women also had experience working as dental assistants, and one woman was an AHW, which may have influenced the findings. However, the involvement of these women enriched the study as they provided insight from both the perspective of a health care and oral health promotion as well as their lived experiences an Aboriginal woman within the community.

## 6. Conclusions

Despite the existing oral health and pregnancy guidelines and a recent focus on maternal oral health, it appears that the oral health needs among some Aboriginal women during pregnancy are still not adequately addressed. As oral health may not be prioritised due to the multiple responsibilities of the Aboriginal women, this topic needs to be raised by antenatal care providers like AHWs, who have established trust, to provide tailored and culturally safe oral health support and promote oral health self-efficacy.

## Figures and Tables

**Table 1:** Themes and sub-themes.

Main Theme	Sub-Theme
Theme 1: The priority of oral health during pregnancy	Oral health concerns and behaviours Issues with accessing dental services Knowledge and attitudes towards oral health care Juggling competing priorities
Theme 2: Supporting maternal oral health self-efficacy through relationships	Connection and trust with healthcare providers Seeking information to inform oral health choices Tailoring oral health promotional resources

## Supplementary Materials

The following are available online at https://www.mdpi.com/article/10.3390/ijerph18158061/s1, The topic guide for the interviews can be located in Supplementary Material 1.
