# Supplementary material for: A Qualitative Study Exploring the Experiences and Perspectives of Australian Aboriginal Women on Oral Health during Pregnancy
Source: Int J Environ Res Public Health. 2021 Jul 29;18(15):8061. doi: 10.3390/ijerph18158061 (PMC8345349; doi:10.3390/ijerph18158061)
Supplement: Supplementary file 1 [file ijerph-18-08061-s001.zip › ijerph-1195495-supplementary.pdf]

Supplementary Material 1: Interview Prompts

- Background about self and family *(including how closely does the participant identify and relate with Aboriginal and Torres Strait Islander community)*
  - Example: *"Could you tell me about how you identify yourself as an Aboriginal and Torres Strait Islander woman? What mob/community do you identify with?" (if they are able to share)*
- Experiences about your health/your baby's health during pregnancy
  - Example: *Can you tell me about how your pregnancy has been? Have you/did you have any particular concerns?*
- Oral health during pregnancy *(knowledge, importance, past experiences, challenges)*
  - Example: *Have you had any concerns with your oral health/dental with your pregnancies? (If so): How were your concerns managed?*
  - Example: *What do you know/ what are the sorts of things that you've heard from friends and family about seeing the dentist or taking care of your teeth?*
- Involvement of Aboriginal health staff (or other antenatal care provider) in your oral health care *(tease out relationship between woman and the health worker)*
  - Example: *Have you been involved in a program where Aboriginal Health Workers support you during pregnancy?*
- Other comments/questions
